# Supplementary material for: Investigation of Planckian behavior in a high-conductivity oxide: PdCrO2
Source: Proc Natl Acad Sci U S A. 2023 Aug 28;120(36):e2307334120. doi: 10.1073/pnas.2307334120 (PMC10483643; doi:10.1073/pnas.2307334120)
Supplement: Supplementary file 1 — Appendix 01 (PDF) [file pnas.2307334120.sapp.pdf]

Supporting Information for

Investigation of Planckian behavior in a high-conductivity oxide: $PdCrO_2$

Elina Zhakinaa, Ramzy Daou, Antoine Maignan, Philippa H. McGuinness, Markus König, Helge Rosner, Seo-Jin Kim, Seunghyun Khim, Romain Grasset, Marcin Konczykowski, Evyatar Tulipman, Juan Felipe Mendez-Valderrama, Debanjan Chowdhury, Erez Bergd, and Andrew P. Mackenzie

Elina Zhakina.
E-mail: zhakina@cpfs.mpg.de

This PDF file includes:

Supporting text
Figs. S1 to S3
Table S1
SI References

Supporting Information Text

1. Supporting information for density functional calculations

For non-magnetic $PdCoO_2$, the calculated lattice parameters and cell volume are in good agreement with the experimental observation. As often observed empirically in transition metal oxide compounds, the LDA slightly underestimates the bond lengths (and therefore the lattice parameters), whereas GGA leads to slight overestimates. However, an average of both approximations leads to error compensation and a result that is within 1% of the experimental value (DFT: 122.0 Å^3 vs. Exp.: 123.1 Å^3). This applies also for the calculated bulk modulus (DFT: 221.2 GPa vs. Exp.: 224.0 GPa). This good agreement between the calculated and the experimental lattice properties also provides confidence in the calculated phonon spectra and specific heat.

In contrast to $PdCoO_2$, a (hypothetical) non-magnetic calculation for $PdCrO_2$ leads to a strong underestimate of the calculated lattice parameters and cell volume. Whereas the calculated cell volume of both sister compounds is essentially identical, the experimental value for the Cr compound is about 10% larger. This clearly indicates the importance of the local spin polarization for Cr^{3+} for a more accurate description of the lattice properties. Indeed, a spin polarized calculation (with ferromagnetic inter-site order) yields an increase of the (LDA-GGA averaged) cell volume of about 10%, resulting in very good agreement with the experimental value (DFT: 133.2 Å^3 vs. Exp.: 133.8 Å^3). This is directly connected with a strong softening for the bulk modulus by about 15% compared to the non-magnetic calculation. There is no known experimental value for the bulk modulus of $PdCrO_2$; we predict $B = 181$ GPa from our spin-polarized calculations. This is more than 20% smaller than that of $PdCoO_2$. Since the energy scale for the on-site spin polarization is about an order of magnitude larger than that of the inter-site coupling, we expect the specific inter-site magnetic order to have only a small influence on lattice parameters and bulk modulus. From the very good agreement between the calculated lattice properties (lattice parameters, cell volume, bulk modulus) for both compounds with the available experimental data we expect also a good agreement for other related lattice properties like the phonon spectra and the derived vibrational parts of the specific heat and the entropy (compare figure 5b main text and figures S1 - S3 of the Supporting Information).

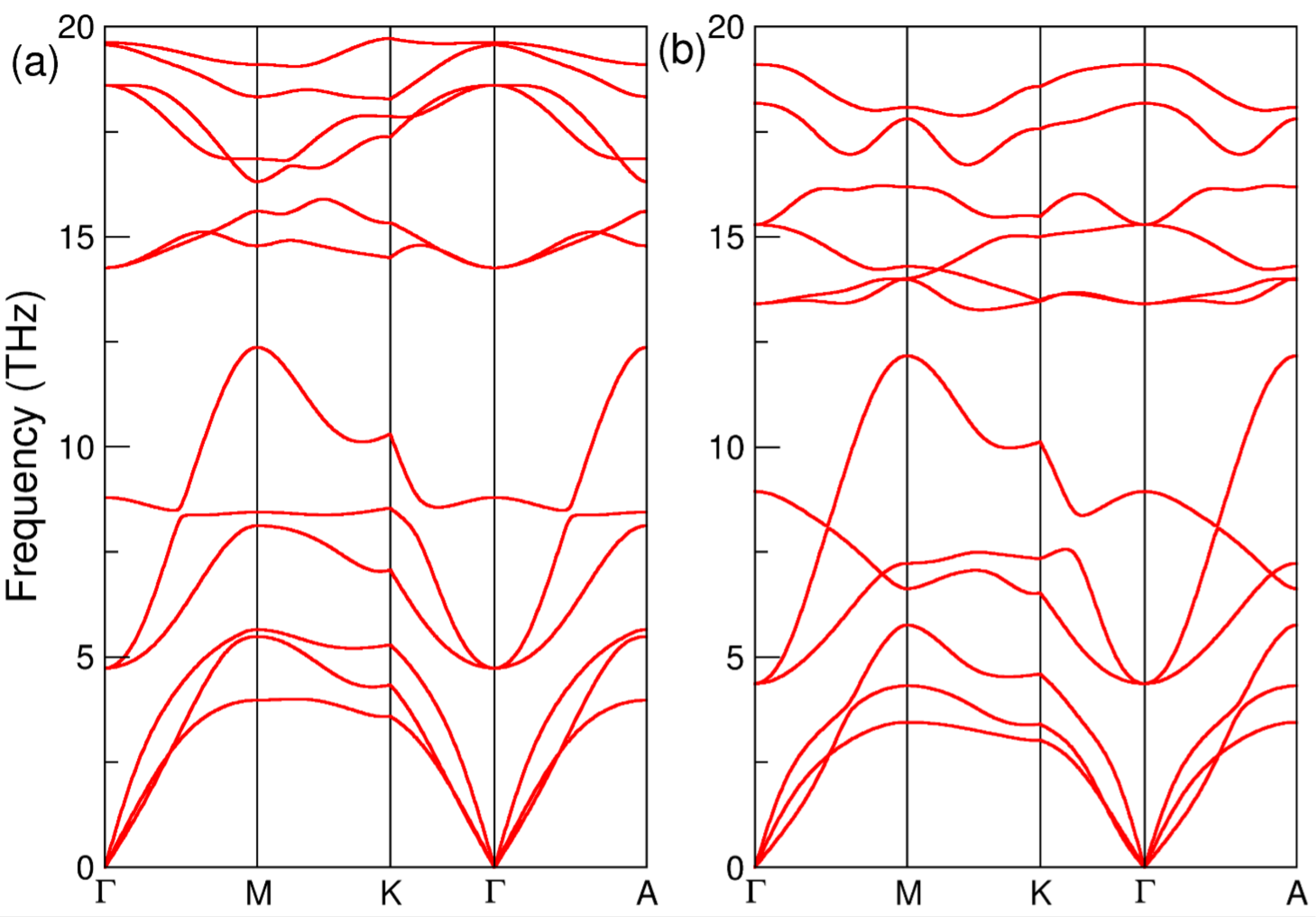

Fig. S1. Calculated phonon dispersions along the standard hexagonal band path of (a) $PdCoO_2$ (non-magnetic calculation) and (b) $PdCrO_2$ (spin polarized calculation) applying GGA as the exchange correlation potential. The spectra of both compounds are rather similar, as expected given their structural similarity. However, the frequencies in the Cr compound are slightly smaller, in particular for the high energy part of the spectrum.

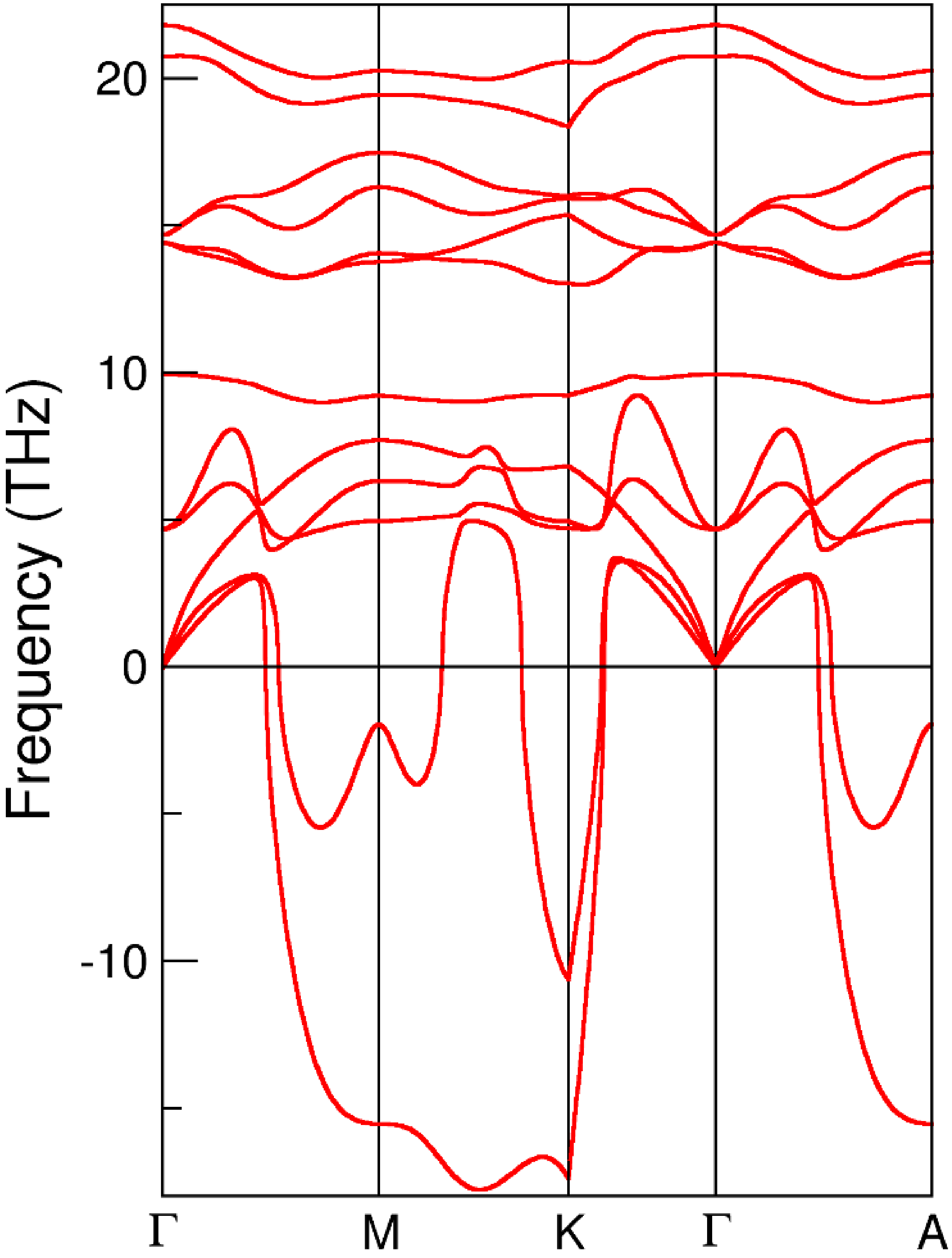

Fig. S2. Calculated phonon dispersions of non-spin-polarized $PdCrO_2$ (LDA, a converged GGA calculation could not be obtained). The dispersion of the non-spin-polarized $PdCrO_2$ shows imaginary frequencies, which implies the instability of the lattice without Cr spin polarization.

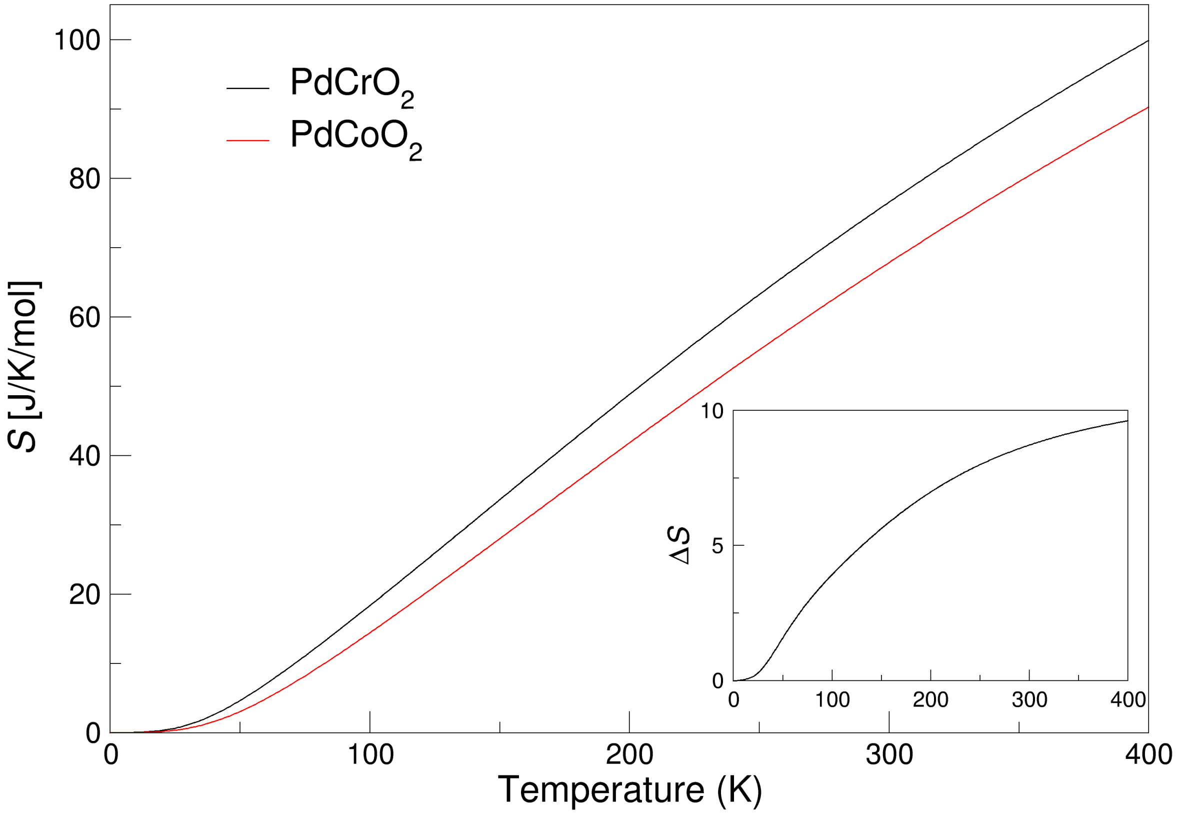

Fig. S3. Calculated vibrational entropy of $PdCrO_2$ (black solid line) and $PdCoO_2$ (red solid line) applying GGA for the exchange correlation functional. For $PdCrO_2$ the inclusion of spin polarization was required to obtain a stable lattice. Inset: Calculated vibrational entropy difference ΔS = $S_{\mathrm{PdCrO_2}}$ - $S_{\mathrm{PdCoO_2}}$ between $PdCrO_2$ and $PdCoO_2$.

Table S1. Calculated lattice parameters and bulk moduli of $PdTO_2$ (T = Co, Cr) applying different density functionals (LDA vs. GGA). The bulk moduli were obtained by fitting the total energies of different volumes to the Birch-Murnaghan equation. Our results are compared to theoretical and experimental literature data. "NM" refers to non-magnetic calculations, "SP" to calculations including spin polarization, in the present case with ferromagnetic order. The dominant change compared to the non-magnetic case, however, originates from the local spin polarization and not from the specific type of inter-site order.

Compound	method	V_0 [Å^3]	a [Å]	c/a	B [GPa]
$PdCoO_2$ (NM)	GGA	126.53	2.86	6.23	194.51
	LDA	117.43	2.79	6.27	247.82
ref. (1)	GGA	128.32	2.86	6.31	206.86
	LDA	119.02	2.79	6.35	264.75
ref. (2)	Exp.	123.06	2.83	6.27	224.00
$PdCrO_2$ (NM)	GGA	126.60	2.76	6.94	180.82
	LDA	117.19	2.68	7.02	238.85
$PdCrO_2$ (SP)	GGA	138.65	2.97	6.09	167.40
	LDA	127.89	2.88	6.19	194.50
ref. (3)	Exp.	133.82	2.92	6.19	-

References

1. S Kumar, H Gupta, Karandeep, First principles study of structural, bonding and vibrational properties of $PtCoO_2$, $PdCoO_2$ and $PdRhO_2$ metallic delafossites. *J. Phys. Chem. Solids* **74**, 305–310 (2013).
2. M Hasegawa, M Tanaka, T Yagi, H Takei, A Inoue, Compression behavior of the delafossite-type metallic oxide $PdCoO_2$ below 10 GPa. *Solid state communications* **128**, 303–307 (2003).
3. V Sunko, et al., Probing spin correlations using angle-resolved photoemission in a coupled metallic/Mott insulator system. *Sci. advances* **6**, eaaz0611 (2020).
